# Supplementary material for: Oxidation-Induced Detachment of Ruthenoarene Units and Oxygen Insertion in Bis-Pd(II) Hexaphyrin π-Ruthenium Complexes
Source: Molecules. 2020 Jun 15;25(12):2753. doi: 10.3390/molecules25122753 (PMC7357109; doi:10.3390/molecules25122753)
Supplement: Supplementary file 1 [file molecules-25-02753-s001.pdf]

Supplementary Material

for

Oxidation-Induced Detachment of Ruthenoarene Unit and Oxygen Insertion in Bis-Pd(II) Hexaphyrin π-Ruthenium Complexes

Akito Nakai,[1] Takayuki Tanaka,[1,*] and Atsuhiro Osuka [1,*]

[1] Department of Chemistry, Graduate School of Science, Kyoto University; nakai@shuyu.kuchem.kyoto-u.ac.jp (A.N.); taka@kuchem.kyoto-u.ac.jp (T.T.); osuka@kuchem.kyoto-u.ac.jp (A.O.)

* Correspondence: taka@kuchem.kyoto-u.ac.jp; Tel.: +81-(0)75-753-4007

Contents

1. NMR spectra
2. HR mass spectra
3. Absorption spectra
4. Electrochemical properties
5. Crystallographic data
6. Resonance contributors and HOSE values
7. DFT calculations
8. References

1. NMR spectra

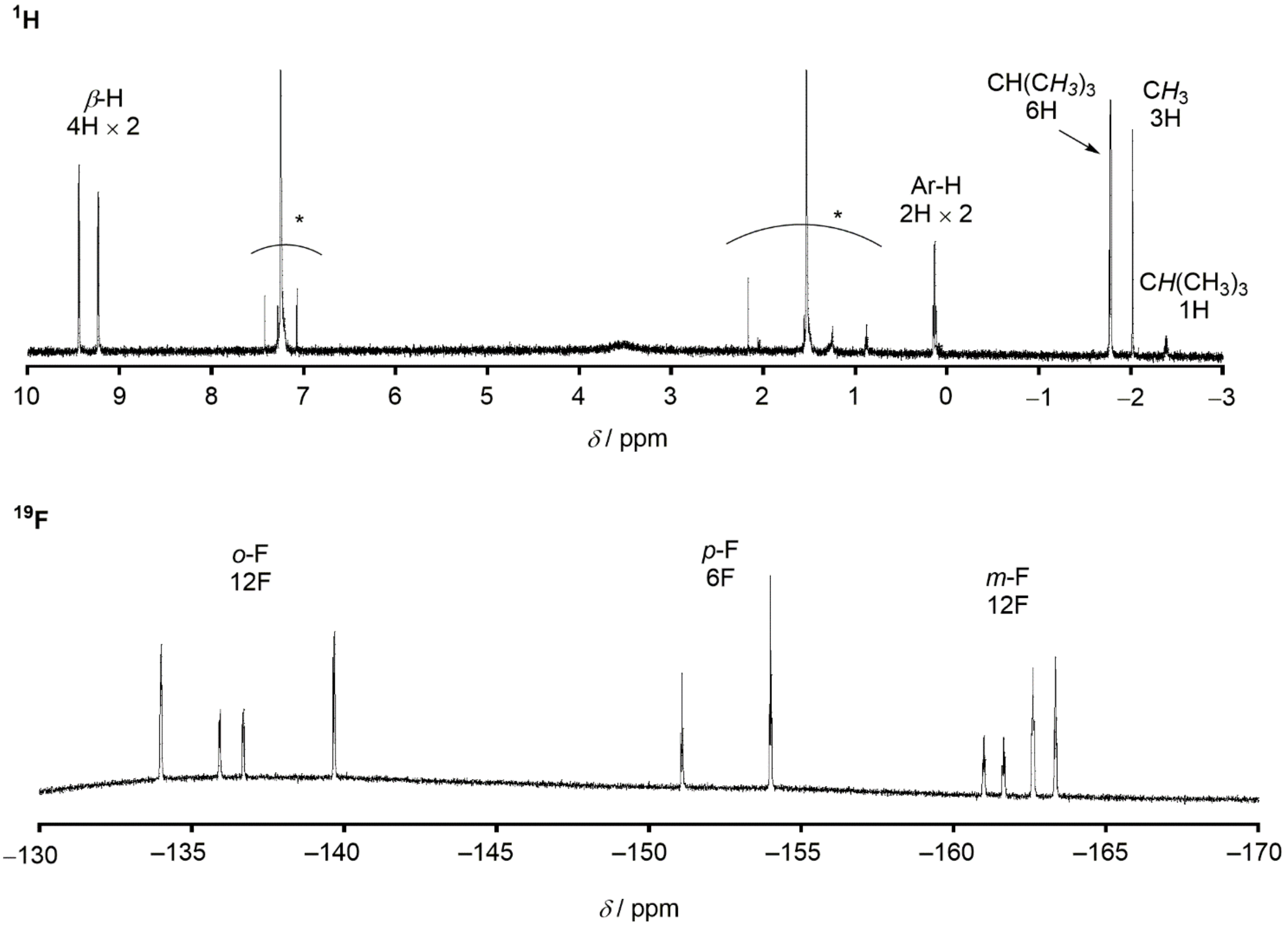

Figure S1. ^{1}H and ^{19}F NMR spectra of **4** in $CDCl_3$ at room temperature. Peak marked with * are due to residual solvents.

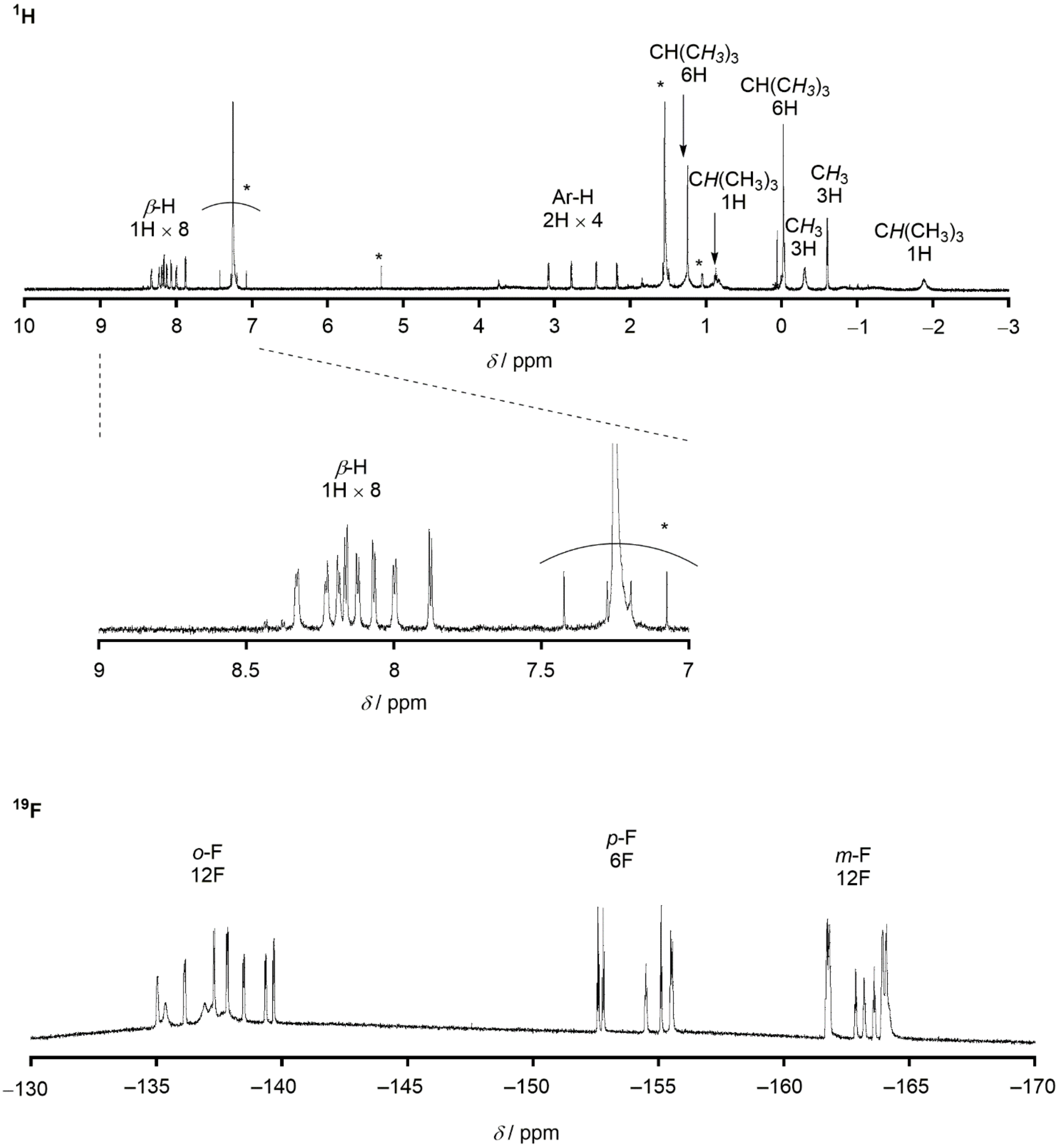

Figure S2. ^{1}H and ^{19}F NMR spectra of **6** in $CDCl_3$ at room temperature. Peak marked with * are due to residual solvents and impurities.

2. HR mass spectra

(a)

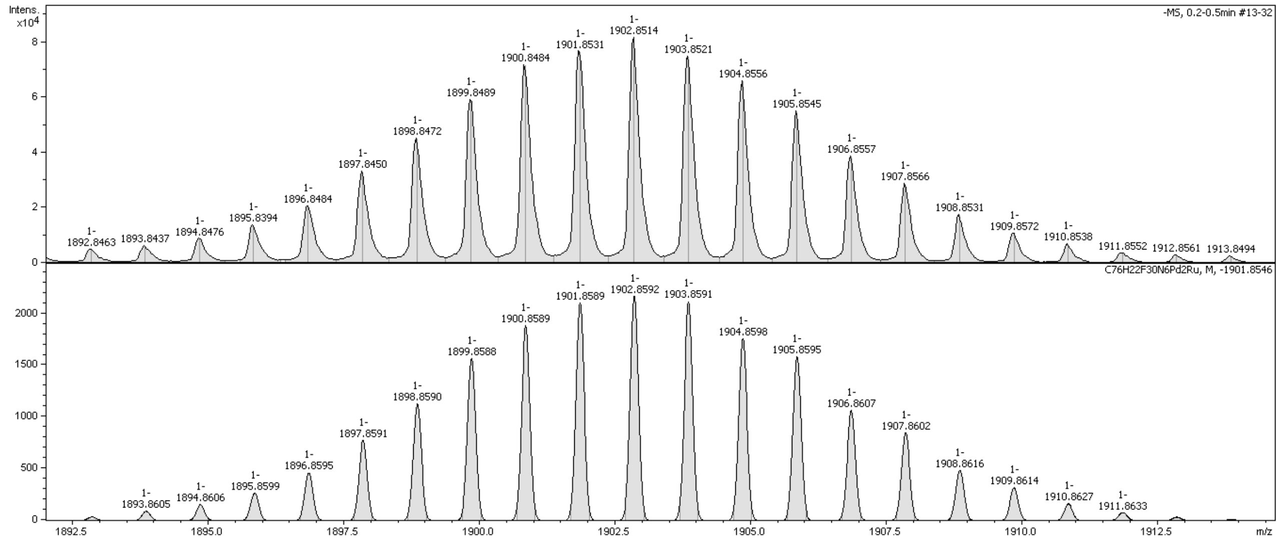

(b)

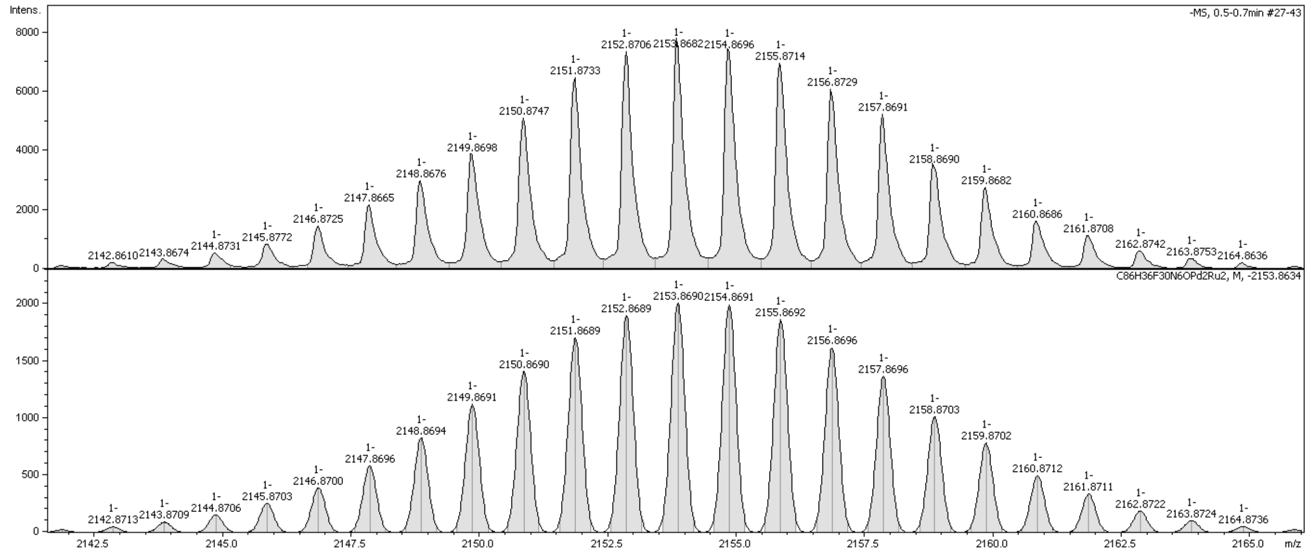

Figure S3. Observed (top) and simulated (bottom) HR APCI-TOF-MS spectra of (a) **4** and (b) **6**.

3. Absorption spectra

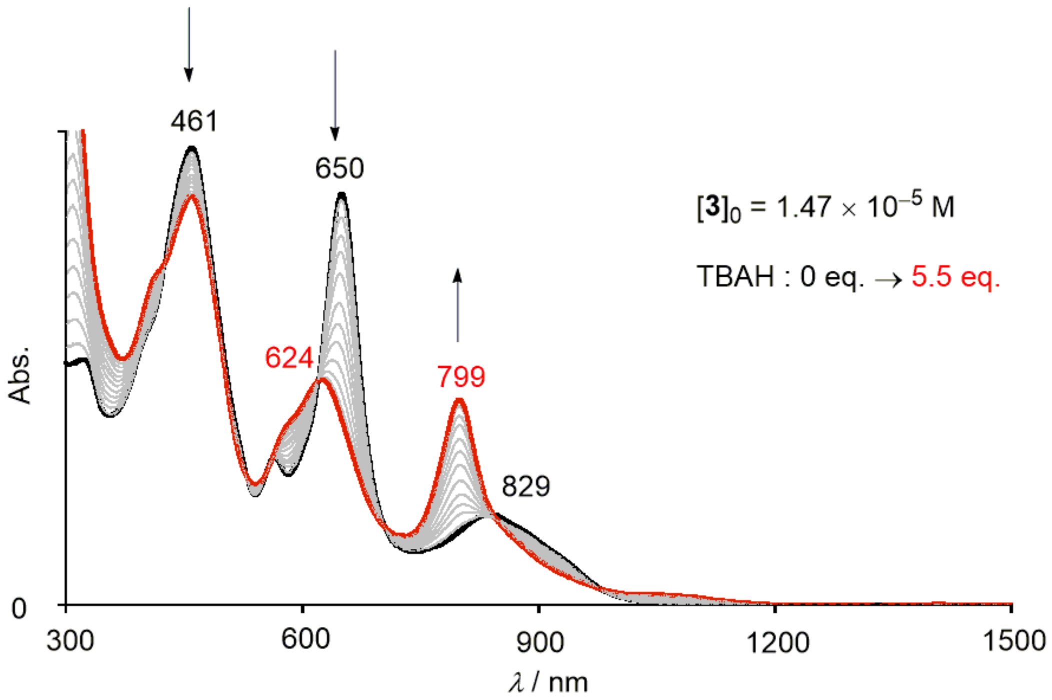

Figure S4. Absorption spectral changes of titration experiments upon addition of TBAH to the solution of **3** in CH_2Cl_2.

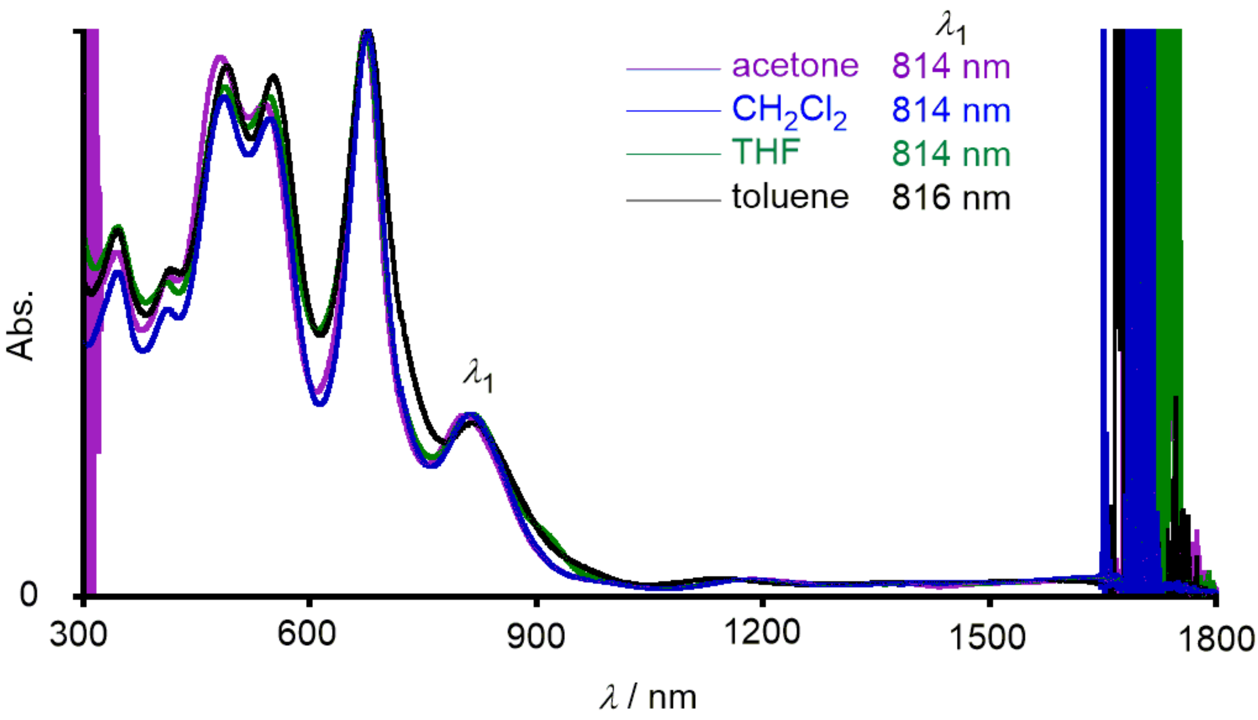

Figure S5. Absorption spectra of **4** in four solvents indicating almost no solvent polarity effect.

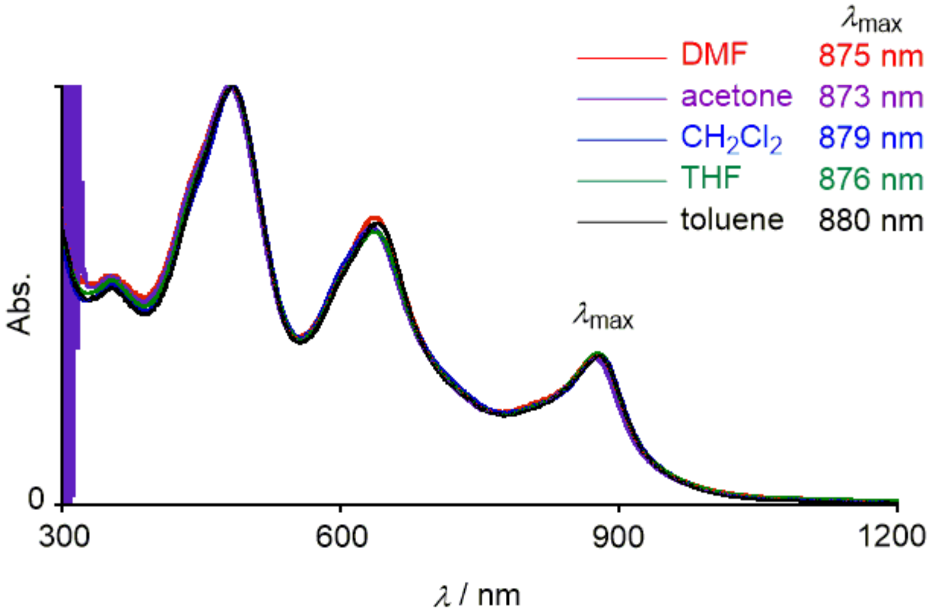

Figure S6. Absorption spectra of **6** in five solvents indicating almost no solvent polarity effect.

4. Electrochemical properties

(a)

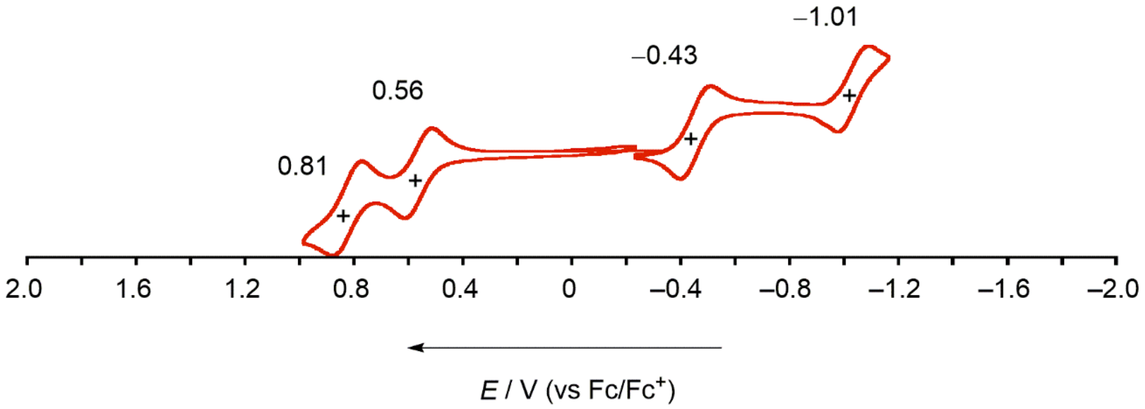

(b)

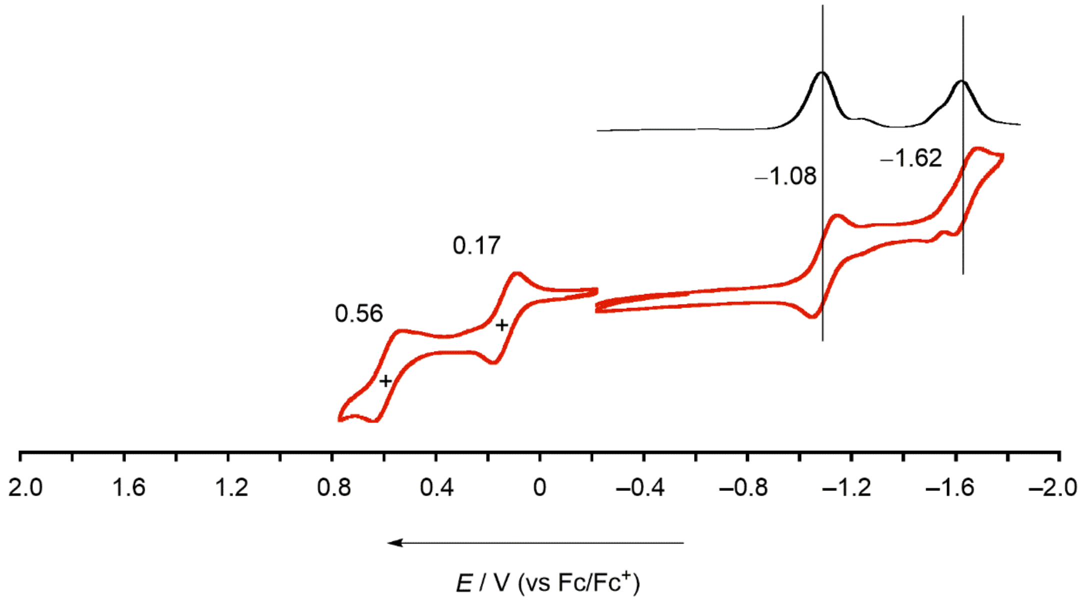

Figure S7. Cyclic voltammograms of (a) **4**, and (b) **6**. Differential pulse voltammograms of **7** and are also shown. Measurement conditions: solvent: CH_2Cl_2; scan rate: 0.05 V/s; working electrode: Pt; reference electrode: Ag/$AgNO_3$; counter electrode: Pt; electrolyte: 0.1 M *n*Bu_4NPF_6.

5. Crystallographic data

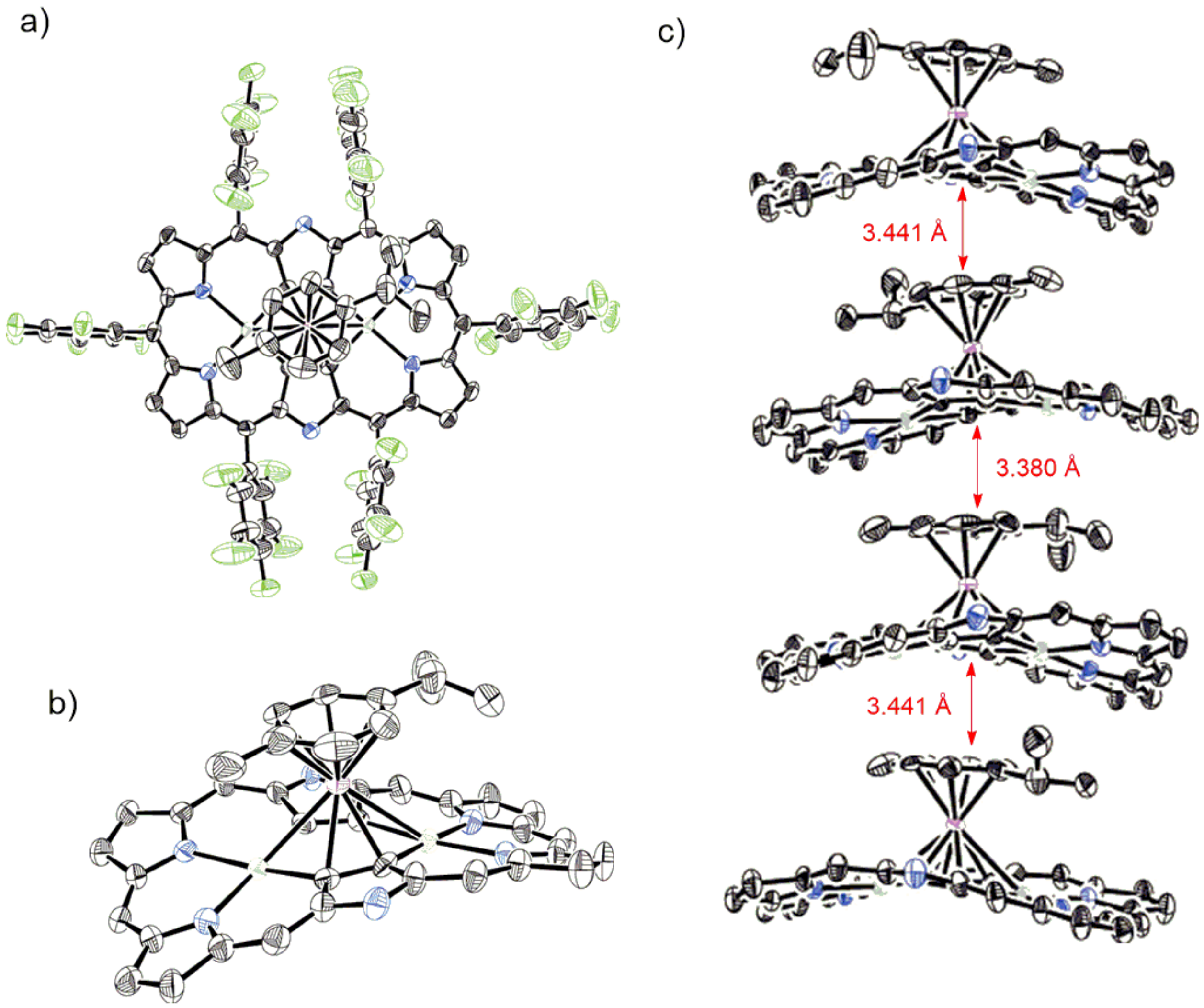

Figure S8. X-Ray crystal structure of **4** (a) top view, (b) perspective view, and (c) packing view. The thermal ellipsoids are 50% probability. For (a) and (b), one of two independent molecules is shown. Solvent molecules, pentafluorophenyl group (perspective and packing view) and hydrogen atoms are omitted for clarity.

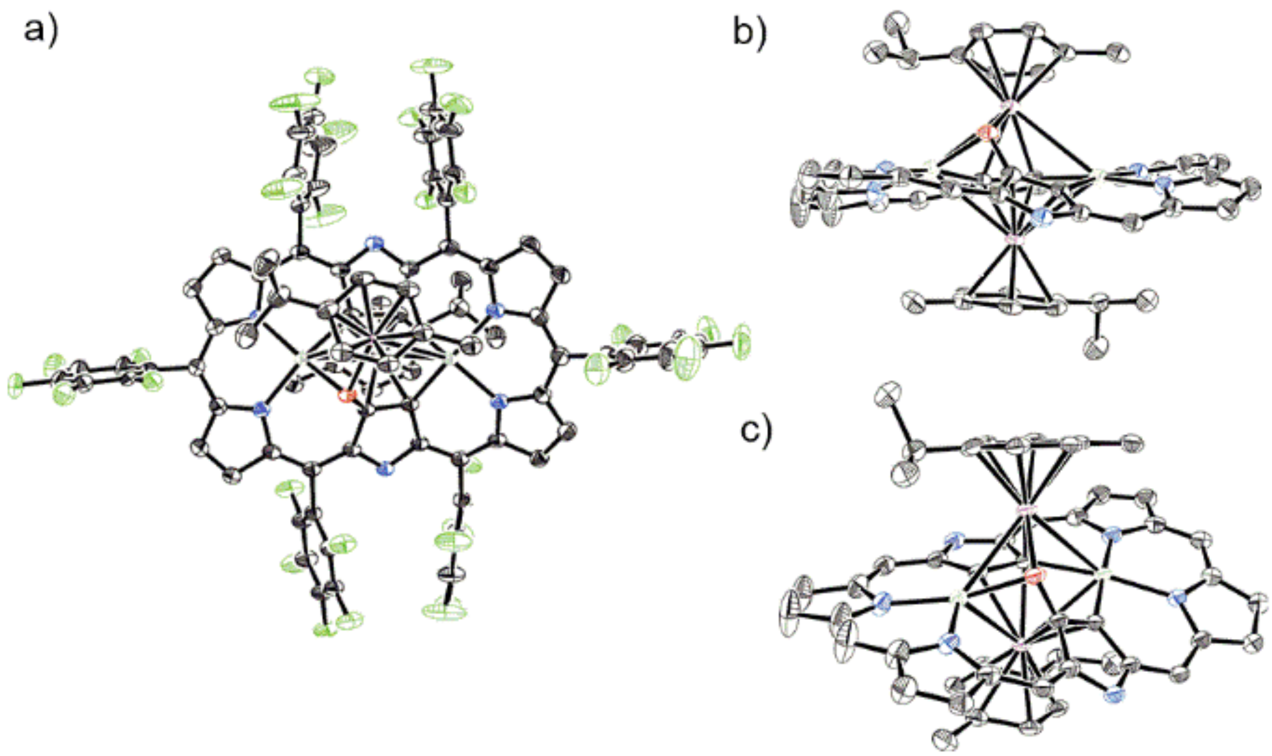

Figure S9. X-Ray crystal structure of **6** (a) top view, (b) side view, and (c) perspective view. The thermal ellipsoids are 50% probability. Solvent molecules, pentafluorophenyl group (side and perspective view) and hydrogen atoms except for NHs are omitted for clarity.

	**4**	**6**
formula	2($C_{76}H_{22}F_{30}N_6Pd_2Ru$), 2($CH_2Cl_2$), C_6H_{14}	$C_{86}H_{36}F_{30}N_6OPd_2Ru_2$, 1.45($CH_2Cl_2$), 2(CO)
solvent	CH_2Cl_2 / *n*-hexane	CH_2Cl_2 / MeOH, H_2O
M_r	4061.76	2333.14
T[K]	93	93
crystal system	orthorhombic	monoclinic
space group	$P2_1\ 2_1\ 2_1$ (No.19)	$P2_1/c$ (No.14)
a [Å]	19.7984(1)	17.9085(1)
b [Å]	26.4804(1)	17.8783(2)
c [Å]	27.2848(1)	26.0682(2)
α [°]	90	90
β [°]	90	103.841(1)
γ [°]	90	90
V [Å^3]	14304.59(10)	8103.99(12)
Z	4	4
ρ_{calcd} [g / cm^3]	1.886	1.912
R_1 [$I > 2\sigma(I)$]	0.0513	0.0347
wR_2 (all data)	0.1470	0.0980
GOF [$I > 2\sigma(I)$]	1.049	1.064
CCDC No.	2005822	2005823

Table S1. Bond lengths (X-ray and DFT calculation) and bond order (DFT) of **4**.

	Bond length (X-ray) [Å]	Bond length (DFT) [Å]	Bond order (DFT)
Pd1-C1	1.988(9)	2.015	0.5979
Pd1-C4	2.014(9)	2.031	0.5544
Pd1-Ru1	2.8269(8)	2.878	0.1956
Pd2-C2	2.002(9)	2.026	0.5652
Pd2-C3	1.996(8)	2.009	0.6045
Pd2-Ru1	2.8417(8)	2.937	0.1883
C1-C2	1.410(13)	1.394	1.4605
C1-Ru1	2.306(8)	2.367	0.3532
C2-Ru1	2.219(8)	2.301	0.4032
C3-C4	1.380(13)	1.394	1.4628
C3-Ru1	2.348(8)	2.420	0.3274
C4-Ru1	2.215(8)	2.286	0.4224

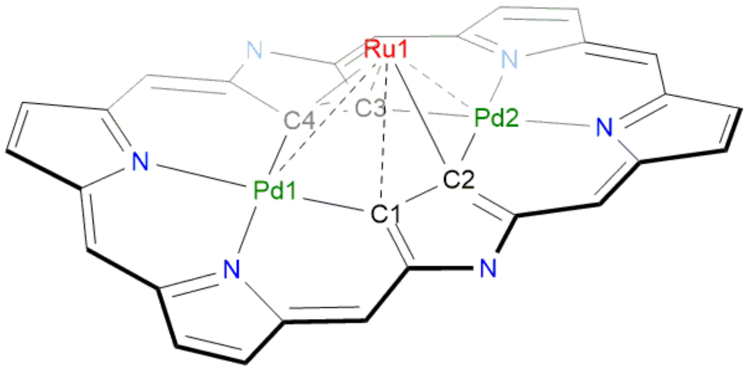

Table S2. Bond lengths (X-ray and DFT calculation) and bond order (DFT) of **6**.

	Bond length (X-ray) [Å]	Bond length (DFT) [Å]	Bond order (DFT)
Pd1-C1	2.552(3)	2.591	0.1179
Pd1-C4	2.057(3)	2.080	0.4421
Pd1-O1	2.014(2)	2.039	0.2628
Pd1-Ru1	2.9548(5)	2.947	0.2351
Pd1-Ru2	2.9651(6)	3.035	0.2453
Pd2-C2	1.961(3)	1.980	0.5061
Pd2-C3	2.099(3)	2.134	0.4516
Pd2-Ru1	3.1146(6)	3.216	0.2357
Pd2-Ru2	2.8568(5)	2.866	0.2848
C1-C2	1.404(4)	1.410	1.1260
C1-O1	1.355(4)	1.337	0.8766
C1-Ru1	3.036(3)	3.102	0.0109
C1-Ru2	2.396(4)	2.507	0.3298
C2-Ru1	3.367(3)	3.483	0.0562
C2-Ru2	2.250(4)	2.249	0.4557
C3-C4	1.478(4)	1.464	1.0713
C3-Ru1	2.179(3)	2.179	0.4825
C3-Ru2	2.263(3)	2.402	0.4331
C4-Ru1	2.242(3)	2.314	0.4285
C4-Ru2	2.239(3)	2.296	0.4366
Ru1-O1	2.120(2)	2.145	0.4018

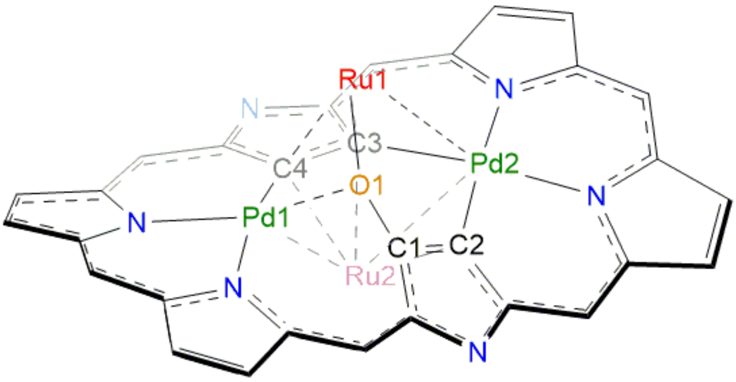

6. Resonance contributors and HOSE values

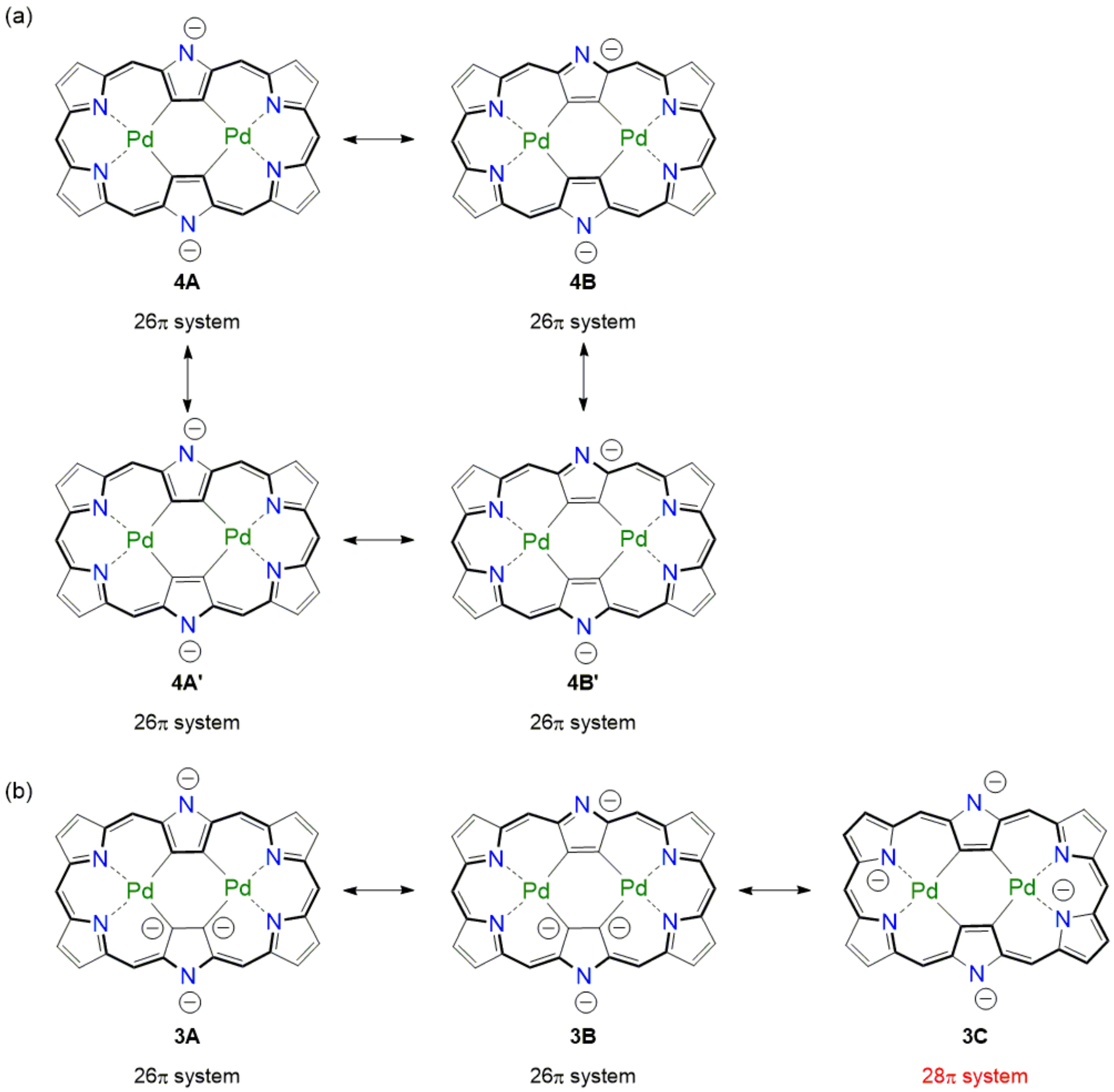

Scheme S1. Plausible resonance contributors of hexaphyrin ligands of (a) **4** and (b) **3**. Effective π-conjugated structures are indicated in bold lines. *meso*-Pentafluorophenyl groups are omitted for clarity.

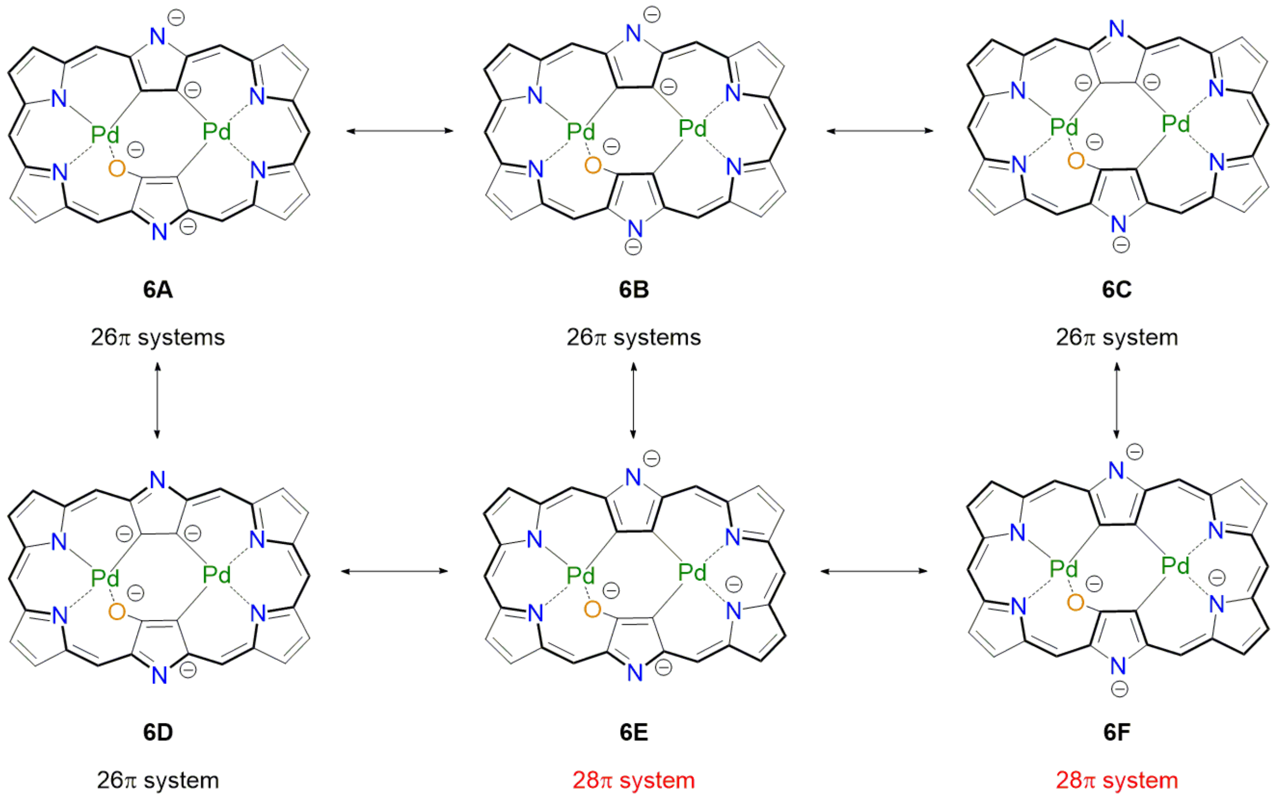

Scheme S2. Plausible resonance contributor of hexaphyrin ligands of **6**. Effective π-conjugated structures are indicated in bold lines. *meso*-Pentafluorophenyl groups are omitted for clarity.

HOSE calculation[S1]

For the calculation of HOSE, the lengths of π bonds in the real molecule were used for the lengths given in the X-ray crystal structures and optimized structures of **4** (for the dianion) and **3** (for the tetraanion).

Table S3. Harmonic oscillator stabilization energies ($HOSE_i$, kcal/mol) and estimated weights (C_i) for canonical structures **4A**, **4B**, **4A′**, **4B′**, **3A**, **3B**, and **3C**.

	$HOSE_i$ (X-ray)	C_i (X-ray)	$HOSE_i$ (DFT)	C_i (DFT)
**4A**	430	0.481	382	0.484
**4B**	399	0.519	358	0.516
**4A′**	508	—	453	—
**4B′**	476	—	430	—
**3A**	427	0.342	444	0.331
**3B**	437	0.334	446	0.329
**3C**	449	0.325	432	0.340

Since **4A** and **4A′**, **4B** and **4B′** have same structure with different conjugated macrocycles, **4A′** and **4B′** are not considered for estimated weights (C_i).

Table S4. Harmonic oscillator stabilization energies ($HOSE_i$, kcal/mol) and estimated weights (C_i) for canonical structures **6A** to **6F**.

	$HOSE_i$ (X-ray)	C_i (X-ray)	$HOSE_i$ (DFT)	C_i (DFT)
**6A**	312	0.189	309	0.189
**6B**	322	0.182	315	0.185
**6C**	350	0.168	344	0.169
**6D**	362	0.162	355	0.164
**6E**	388	0.152	389	0.149
**6F**	401	0.147	402	0.145

Harmonic oscillator stabilization energies were calculated using the method of Krygowski *et al.* using the original parameterization; estimated weights (C_i) were calculated according to the formula

$$C_i = \frac{(\mathrm{HOSE}_i)^{-1}}{\sum_j (\mathrm{HOSE}_j)^{-1}}$$

7. DFT calculations

All calculations were carried out using the *Gaussian 16* program.[S2] All structures were fully optimized without any symmetry restriction. The calculations were performed by the density functional theory (DFT) method with restricted B3LYP (Becke's three-parameter hybrid exchange functionals and the Lee-Yang-Parr correlation functional) level, employing a basis set 6-31G(d) for C, H, N, O, F and LANL2DZ for Pd, Ru. The NICS values and absolute ^{1}H shielding values were obtained with the GIAO method at the B3LYP level, employing a basis set 6-31G(d) for C, H, N, O, F and LANL2DZ for Pd, Ru. The global ring centers for the NICS values were designated at the nonweighted means of the carbon and nitrogen coordinates on the peripheral positions of conjugated macrocycles. In addition, NICS values were also calculated on centers of other local cyclic structures as depicted in the figures. The anisotropy of the induced current density (ACID) calculation was conducted with the CSGT method.[S3] The bond order was conducted with the NBO program.[S4] Oscillator strengths were calculated with the TD-SCF method at the B3LYP level.

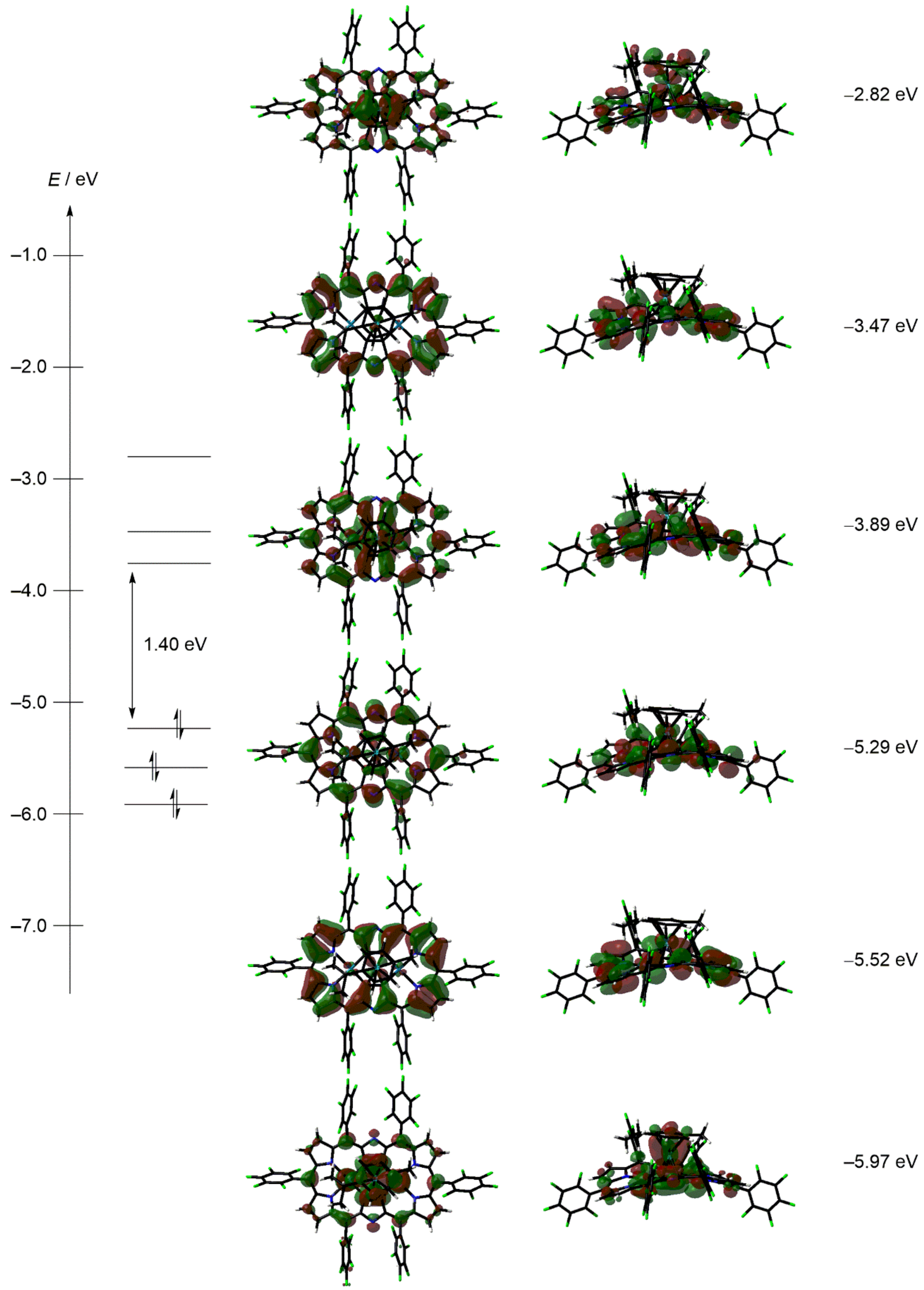

Figure S10. Kohn–Sham orbital diagrams of **4**.

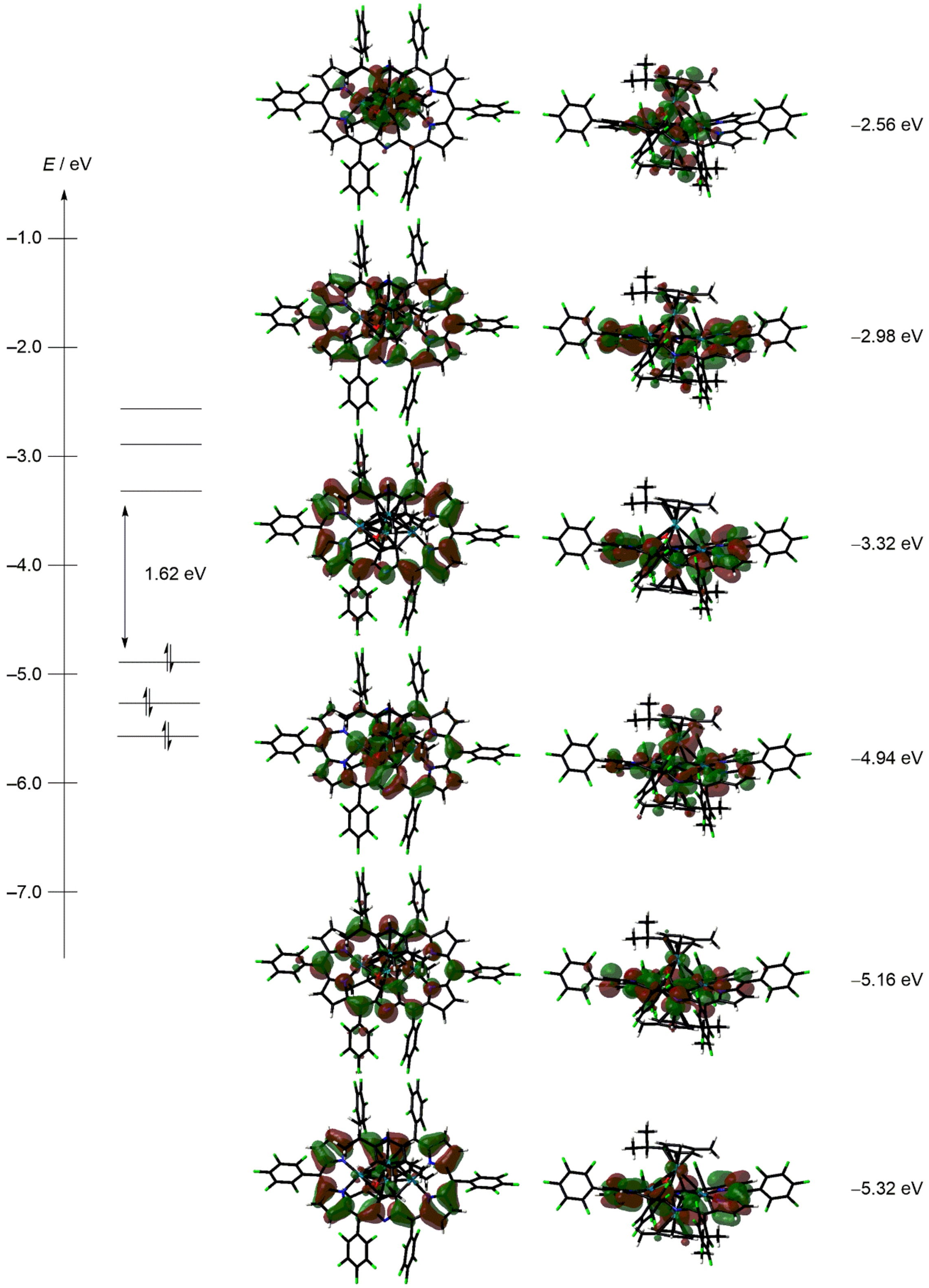

Figure S11. Kohn–Sham orbital diagrams of **6**.

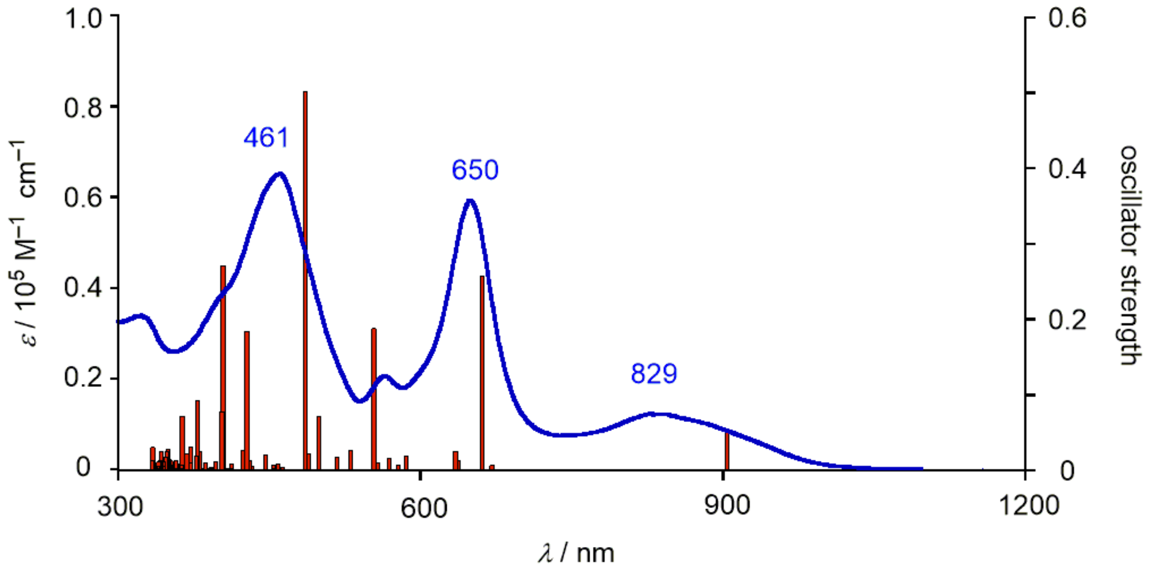

Figure S12. Calculated absorption spectrum of **3** on the basis of optimized structure (red bar), observed absorption spectrum (blue line). Selected oscillator strengths: f = 0.0001 (1011 nm), 0.0505 (904 nm), 0.2581 (661 nm), 0.1880 (554 nm), 0.5017 (486 nm), 0.1846 (427 nm), and 0.2712 (404 nm).

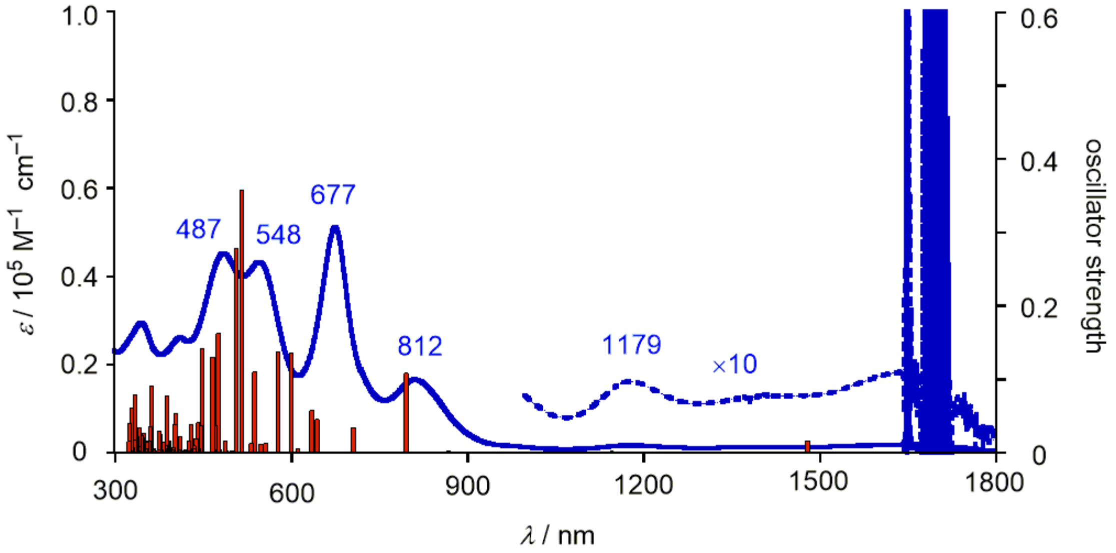

Figure S13. Calculated absorption spectrum of **4** on the basis of optimized structure (red bar), observed absorption spectrum (blue line). Selected oscillator strengths: f = 0.0166 (1479 nm), 0.0025 (1146 nm), 0.1084 (795 nm), 0.1358 (599 nm), 0.1377 (577 nm), 0.3587 (515 nm), and 0.2791 (506 nm).

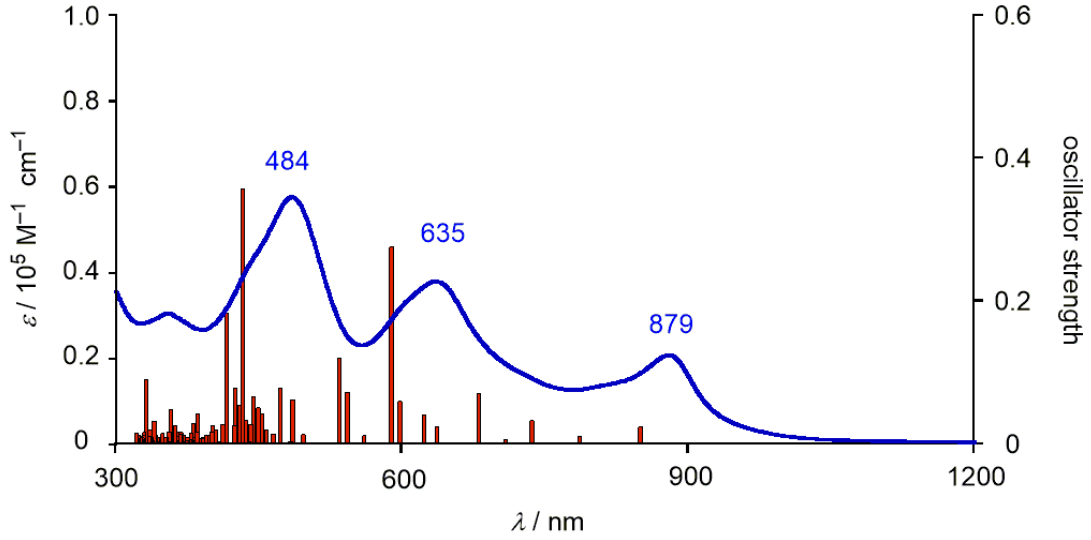

Figure S14. Calculated absorption spectrum of **6** on the basis of optimized structure (red bar), observed absorption spectrum (blue line). Selected oscillator strengths: f = 0.0049 (1074 nm), 0.0235 (851 nm), 0.2755 (590 nm), 0.3568 (433 nm), and 0.1830 (417 nm).

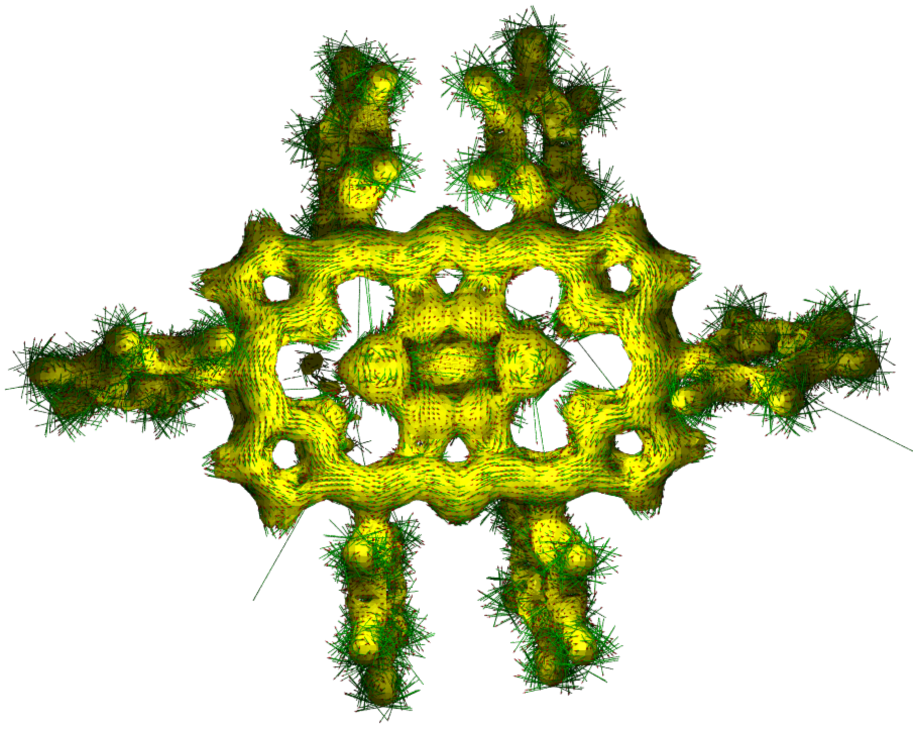

Figure S15. ACID isosurface of **4** at isovalue of 0.05. The external magnetic field was applied in the direction from back of the paper to the surface. The current density vectors plotted onto the ACID isosurface indicate the diatropic ring current.

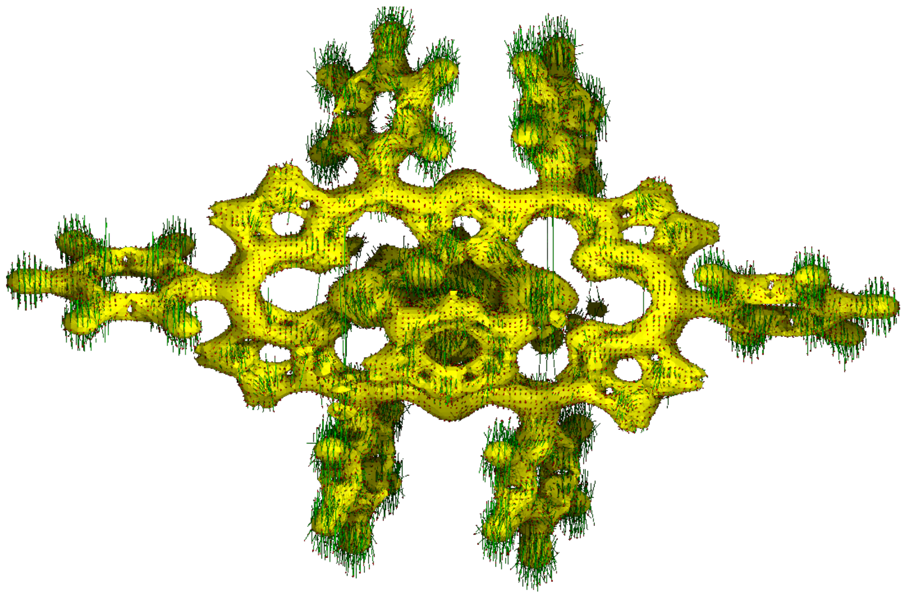

Figure S16. ACID isosurface of **6** at isovalue of 0.05. The external magnetic field was applied in the direction from back of the paper to the surface. The current density vectors plotted onto the ACID isosurface indicate no macrocyclic ring current.

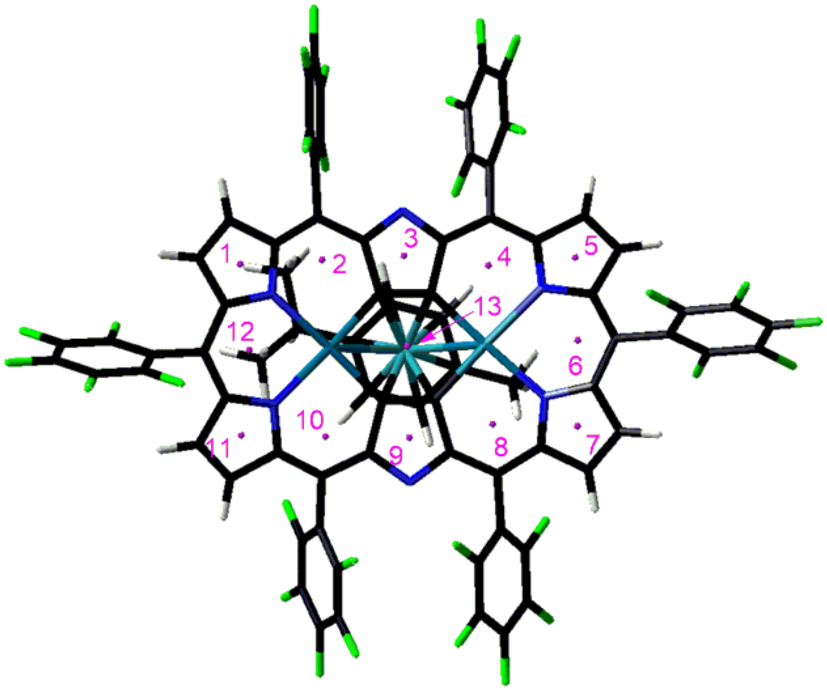

position	NICS (ppm)
1	–2.77
2	–20.45
3	–12.47
4	–17.93
5	–3.73
6	–22.20
7	–2.94
8	–18.04
9	–11.82
10	–19.86
11	–3.56
12	–18.63
13	–24.74

Figure S17. NICS(0) values at selected points of **4**.

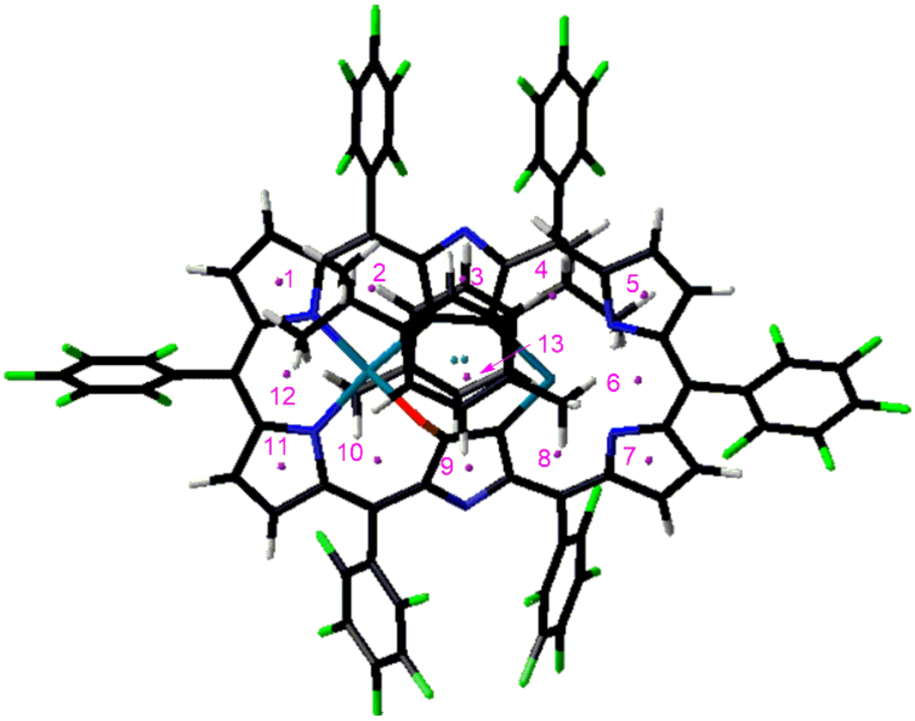

position	NICS (ppm)
1	–0.56
2	–11.57
3	–11.97
4	–10.03
5	–0.51
6	–14.13
7	–0.40
8	–9.00
9	–12.47
10	–10.19
11	–0.36
12	–15.27
13	–29.75

Figure S18. NICS(0) values at selected points of **6**.

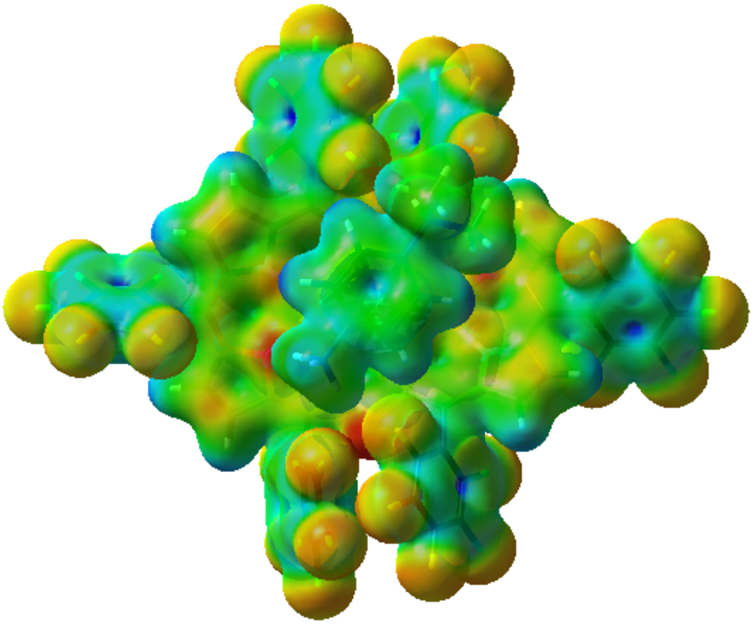

Figure S19. Three-dimensional molecular electrostatic potential (–0.035e Å^{-3}, red and 0.110e Å^{-3}, blue) mapped on the surface of total electron density (–0.01e Å^{-3}) for **3**.

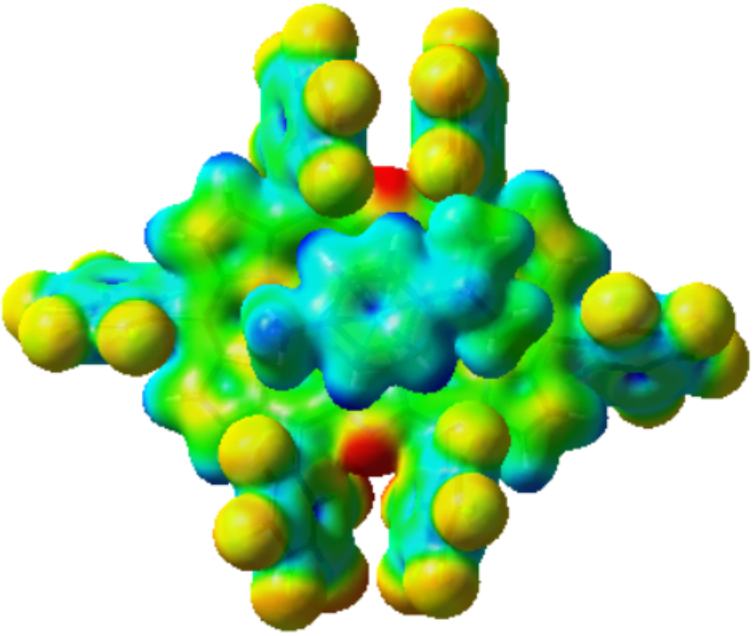

Figure S20. Three-dimensional molecular electrostatic potential (–0.035e $Å^{-3}$, red and 0.110e $Å^{-3}$, blue) mapped on the surface of total electron density (–0.01e $Å^{-3}$) for **4**.

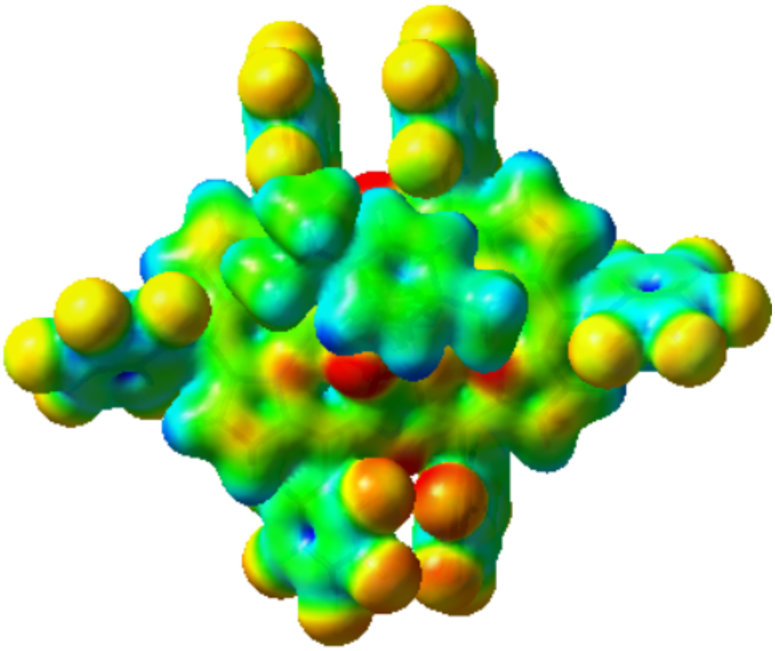

Figure S21. Three-dimensional molecular electrostatic potential (–0.035e $Å^{-3}$, red and 0.110e $Å^{-3}$, blue) mapped on the surface of total electron density (–0.01e $Å^{-3}$) for **6**.

8. References

[S1] Krygowski, T.M.; Szatylowicz, H.; Stasyuk, O.A.; Dominikowska, J.; Palusiak, M. *Chem. Rev.* **2014**, *114*, 6383.

[S2] Gaussian 16, Revision A.03, Frisch, M.J.; Trucks, G.W.; Schlegel, H.B.; Scuseria, G.E.; Robb, M.A.; Cheeseman, J.R.; Scalmani, G.; Barone, V.; Petersson, G.A.; Nakatsuji, H.; Li, X.; Caricato, M.; Marenich, A.V.; Bloino, J.; Janesko, B.G.; Gomperts, R.; Mennucci, B.; Hratchian, H.P.; Ortiz, J.V.; Izmaylov, A.F.; Sonnenberg, J.L.; Williams-Young, D.; Ding, F.; Lipparini, F.; Egidi, F.; Goings, J.; Peng, B.; Petrone, A.; Henderson, T.; Ranasinghe, D.; Zakrzewski, V.G.; Gao, J.; Rega, N.; Zheng, G.; Liang, W.; Hada, M.; Ehara, M.; Toyota, K.; Fukuda, R.; Hasegawa, J.; Ishida, M.; Nakajima, T.; Honda, Y.; Kitao, O.; Nakai, H.; Vreven, T.; Throssell, K.; Montgomery, J.A., Jr.; Peralta, J.E.; Ogliaro, F.; Bearpark, M.J.; Heyd, J.J.; Brothers, E.N.; Kudin, K.N.; Staroverov, V.N.; Keith, T.A.; Kobayashi, R.; Normand, J.; Raghavachari, K.; Rendell, A.P.; Burant, J.C.; Iyengar, S.S.; Tomasi, J.; Cossi, M.; Millam, J.M.; Klene, M.; Adamo, C.; Cammi, R.; Ochterski, J.W.; Martin, R.L.; Morokuma, K.; Farkas, O.; Foresman, J.B.; Fox, D.J. Gaussian, Inc., Wallingford CT, 2016.

[S3] a) Herges, R.; Geuenich, D. *J. Phys. Chem. A* **2001**, *105*, 3214. b) Geuenich, D.; Hess, K.; Köhler, F.; Herges, R. *Chem. Rev.* **2005**, *105*, 3758.

[S4] NBO Version 3.1, Glendening, E.D.; Reed, A.E.; Carpenter, J.E.; Weinhold, F.
